# Significance of research networking for enhancing collaboration and research productivity

**DOI:** 10.3325/cmj.2014.55.181

**Published:** 2014-06

**Authors:** Livia Puljak, Sandor G. Vari

**Affiliations:** 1 University of Split School of Medicine, Department of Anatomy, Histology and Embryology, Split, Croatia; 2 International Research and Innovation Management Program, Cedars-Sinai Medical Center, Los Angeles, CA, USA

International collaboration is growing exponentially (1) and researchers from different institutions and countries increasingly work together as consortia focused around specific research questions. Such consortia are especially valuable for international health research because they offer interdisciplinary expertise and allow recruitment of patients in different settings (2). Establishing research networks and collaborations in the form of non-governmental organizations (NGO) and non-profit, voluntary participants' groups provides the necessary flexibility to adapt to a wide spectrum of arising challenges. It enables shared learning, new research opportunities, establishing new research projects, joint applications for funds, and technology transfer (3). The collaborations increase citations of research manuscripts, especially if there is an international team of authors involved (4). Building research networks is particularly important for Central and Eastern Europe (CEE) countries, which have fragmented scientific community, small research groups, and scarce financing.

Recognizing the value of research networking, Cedars-Sinai Medical Center (CSMC) started building in 2002 a regional research organization in CEE by establishing International Research and Innovation Management program. During the first four years of the program, CSMC established partnerships with research institutions in Croatia, Czech Republic, Hungary, Romania, Slovakia, and Ukraine. CMSC provided training on research and innovation management, technology transfer infrastructure, and management capacity. In 2006, it formed the Regional Cooperation for Health, Science and Technology (RECOOP HST) Consortium with eleven CEE universities and research organizations from the following countries: Croatia, Czech Republic, Hungary, Romania, Slovakia, Ukraine, and the USA (5).

In 2012, CSMC registered with the Consortium the Association for Regional Cooperation in the Fields of Health, Science and Technology at the Court of Debrecen in Hungary. The newly established Association included members from 8 countries, adding Denmark and Poland to the consortium, and involving 14 higher education or research organizations.

The Association promotes creative thinking and helps its members to make decision whether they want to “publish and disclose” or “protect and publish.” Special attention is paid to young scientists and their education on scientific communication and technology transfer. In 2012, it carried out several integrated multidisciplinary, multicenter research studies within the joint RECOOP Life Science Research Platform and formed 18 CSMC RECOOP Research Centers (CRRC) in 7 countries (Croatia, Czech Republic, Hungary, Poland, Romania, Slovakia, and Ukraine) to facilitate the cooperation on translational and clinical research in the field of genomics-proteomics, epigenetics, metagenomics, molecular biology, metabolomics, and nano-biotechnology. Besides that, among its highest priorities is the involvement of medical and PhD students into the CRRCs’ research programs.

RECOOP Life Science Research Platform already completed a number of short term (1-2 years) pilot research studies, which are now being converted to midterm (5 years) projects. The clinical studies are planned to continue for a minimum of 20-30 years and follow up the women, men and newborns registered in the Electronic Data Entry Forms, started in 2011 (6).

The RECOOP HST Association annually organizes the Bridges in Life Sciences Conferences to review the scientific progress in the Association. At the annual meeting, the Scientific Advisory Board selects the top ten young scientists, for whom the Association covers the attendance costs of summer schools and workshops on manuscript writing and intellectual property protection.

RECOOP plays a significant role in the integration of life science research activities in member countries of Visegrad Four European Macro-Region (Czech Republic, Hungary, Poland, and Slovakia) and their neighboring countries (Belarus, Croatia, Romania, and Ukraine). The Association paves the way for GLOBAL Research Programs of the National Institutes of Health and the World Health Organization, and private funds such as Bill & Melinda Gates Foundation and Clinton Foundation (7).

In Croatia, the RECOOP HST Association includes researchers from the J. J. Strossmayer University School of Medicine in Osijek and University of Split School of Medicine. Professor Ana Marušić from the University of Split School of Medicine, who has extensive experience in teaching and studying research methodology (8,9), continuously organizes education programs for young researchers within the RECOOP HST Association. University of Split School of Medicine has proven its capacity for international collaboration by establishing a branch of The Cochrane Collaboration in 2008 (10) and promoting evidence-based medicine in medical education and practice (11,12). This, together with its continuously increasing research output (13,14), is making the School a desirable partner in international networking scheme.

Since CEE countries are considered a scientific periphery, there must be an even stronger impetus to increase their participation in international research consortia. Sustained engagement in training programs and joint applications to research funding can stimulate research network development (15). RECOOP HST Association is a platform that offers such research networking opportunities. Benefits of participation in such consortium can be fully appreciated in the long-term, but its positive impact is already visible, judging by the number of joint publications and research proposals that are being developed.

As part of the ongoing process of European integration (16), the RECOOP HST Association contributes to the establishment of the “European knowledge society” and increases the competitiveness of CEE. Enhancing international collaboration, possibly using RECOOP HST Association as a model, should be the goal of every researcher and research institution, because such collaboration enables capacity building and offers multiple opportunities to surpass limitations that arise within a single institution and due to scarce resources.
